# C-Phycocyanin protects against mitochondrial dysfunction and oxidative stress in parthenogenetic porcine embryos

**DOI:** 10.1038/s41598-017-17287-0

**Published:** 2017-12-05

**Authors:** Ying-Jie Niu, Wenjun Zhou, Jing Guo, Zheng-Wen Nie, Kyung-Tae Shin, Nam-Hyung Kim, Wen-Fa Lv, Xiang-Shun Cui

**Affiliations:** 1 Department of Animal Science, Chungbuk National University, Cheongju, South Korea; 2 College of Animal Science and Technology, Jilin Agricultural University, Changchun, China

## Abstract

C-Phycocyanin (CP) is a biliprotein enriched in blue-green algae that is known to possess antioxidant, anti-apoptosis, anti-inflammatory, and radical-scavenging properties in somatic cells. However, the protective effect of CP on porcine embryo developmental competence *in vitro* remains unclear. In the present study, we investigated the effect of CP on the development of early porcine embryos as well as its underlying mechanisms. Different concentrations of CP (2, 5, 8, 10 μg/mL) were added to porcine zygote medium 5 during *in vitro* culture. The results showed that 5 μg/mL CP significantly increased blastocyst formation and hatching rate. Blastocyst formation and quality were significantly increased in the 50 μM H_2_O_2_ treatment group following 5 μg/mL CP addition. CP prevented the H_2_O_2_-induced compromise of mitochondrial membrane potential, release of cytochrome c from the mitochondria, and reactive oxygen species generation. Furthermore, apoptosis, DNA damage level, and autophagy in the blastocysts were attenuated by supplementation of CP in the H_2_O_2_-induced oxidative injury group compared to in controls. These results suggest that CP has beneficial effects on the development of porcine parthenotes by attenuating mitochondrial dysfunction and oxidative stress.

## Introduction

Pigs are important for both agricultural production and biomedical research^1^. *In vitro*-produced porcine embryos are extensively used for studying the mechanism of embryonic development and animal reproductive technologies, including cryopreservation, cloning, and transgenesis^2^. The micromilieu surrounding preimplantation embryos during *in vitro* culture is important for the developmental competence of the embryos. Embryos produced *in vitro* show some differences from *in vivo*-derived embryos because when embryos are manipulated and cultured under *in vitro* environmental conditions, exposure to light, greater oxygen stress and culture medium composition has been correlated with oxidative stress generated by the production of reactive oxygen species (ROS)^3,4^. Oxidative stress brings about a number of types of embryo damage. ROS can pass through cell membrane and induce impairment of nucleic acids and lipids proteins. These alterations have numerous effects, including mitochondrial dysfunction, ATP depletion, apoptosis and embryo arrest^3^. Therefore, the use of substances with antioxidant properties during the *in vitro* production of embryos may prevent excessive ROS production and improve embryo developmental competence^5,6^.

C-Phycocyanin (CP) is a major biliprotein found in blue-green algae, such as *Spirulina platensis* ^7^. Numerous beneficial effects of CP have been demonstrated, including antioxidant, anti-apoptosis, anti-inflammatory, and radical-scavenging properties in somatic cells^7–10^. Owing to its health benefits, CP has been used as a blue colorant for food, cosmetics and pharmaceutical industries^11^. Previous studies have shown that CP inhibits ROS production, reverses caspase-3 activity, reduces apoptosis cell population, and prevent mitochondrial dysfunction^12^. A previous study also showed that D-galactose-induced compromise of female reproductive capability can be partially rescued by the antioxidant and anti-apoptosis effects of CP^13^. However, the effect of CP on embryo development during *in vitro* culture has not been examined.

H_2_O_2_ can affect cell viability by disrupting the biological membrane and homeostasis of extracellular matrix^14,15^. Thus, H_2_O_2_ is used to imitate the situation of oxidative stress to examine the potency of antioxidants in alleviating cellular injury^8^. In this study, we first detected the dose-effect of CP on early porcine embryos cultured *in vitro*. Next, the antioxidative effect of CP on porcine embryo development was examined after pre-treatment with H_2_O_2_.

## Results

### Effects of treatment with various concentrations of CP on *in vitro* development of porcine embryos

**Figure 1:**
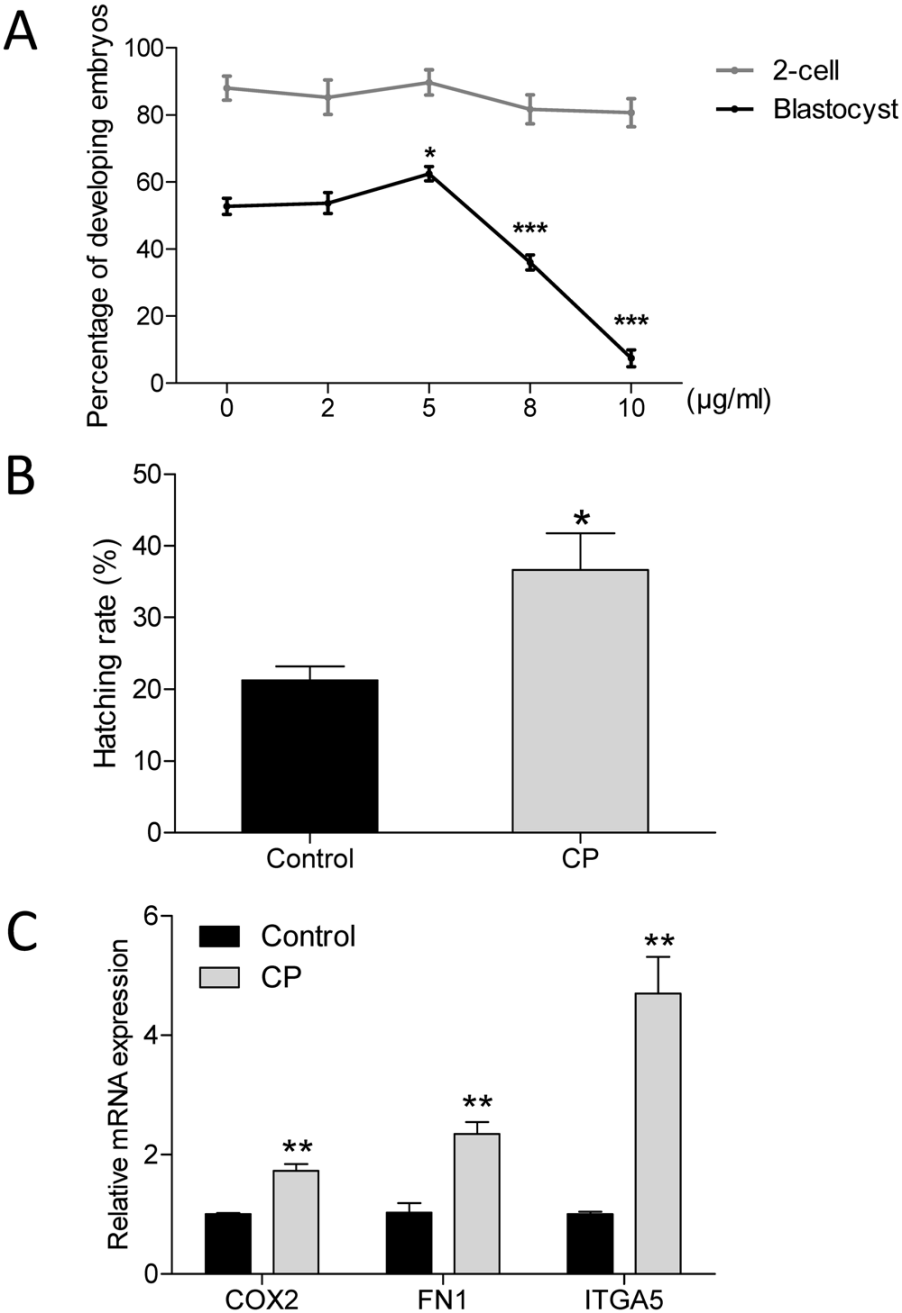
Effects of CP on *in vitro* development of parthenogenetic porcine embryos. (**A**) Cleavage and blastocyst formation of porcine embryos cultured in the presence of CP at different concentrations. (**B**) Percentage of blastocyst hatching in the presence or absence of 5 µg/mL CP in the culture medium. (**C**) Relative expression of hatching-related genes in blastocysts. Data are expressed as the mean ± SEM. In addition, *p < 0.05, **p < 0.01, and ***p < 0.001 indicate significant differences between different groups. The experiments were replicated three times with 612 embryos.

After parthenogenetic activation, one-cell-stage parthenotes were cultured with various concentrations of CP. Figure 1A showed that cleavage was not affected by addition of 2, 5, 8, or 10 μg/mL CP (p > 0.05). Blastocyst formation was also not affected by addition of 2 μg/mL CP (p > 0.05). In addition, addition of 5 μg/mL CP significantly increased the blastocyst rate compared to in other groups (p < 0.05), while high doses of CP (8 and 10 μg/mL) negatively affected blastocyst formation (p < 0.001). Addition of 5 μg/mL CP significantly increased the hatching rate of blastocysts compared to controls (Fig. 1B, p < 0.05). The mRNA expression of hatching-related genes, *COX2*, *FN1*, and *ITGA5*, significantly increased in CP group (Fig. 1C, p < 0.01). Thus, the concentration of CP used in subsequent experiments was 5 μg/mL.

**Figure 2:**
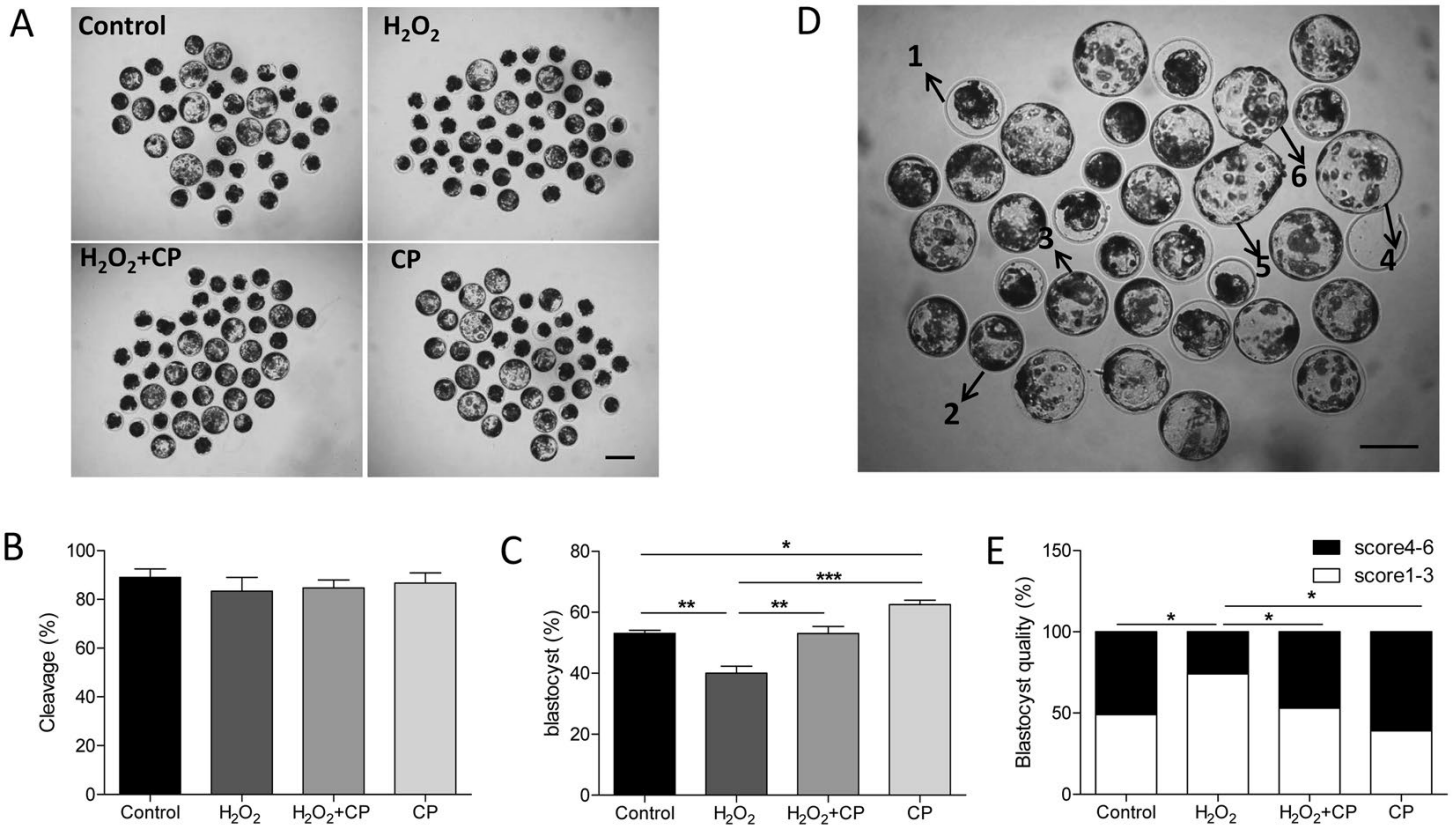
Addition of CP promoted early porcine embryo development in present of H_2_O_2_. Images (**A**), cleavage (**B**), and blastocyst formation (**C**) of embryos in control, H_2_O_2_, H_2_O_2_ + CP, and CP groups. (**D**) Blastocysts were graded on a scale from 1 to 6 as illustrated. Different scores are indicated by the respective numbers. (**E**) Blastocyst quality was examined in the control, H_2_O_2_, H_2_O_2_ + CP, and CP groups. Data are expressed as the mean ± SEM. In addition, *p < 0.05, **p < 0.01, and ***p < 0.001 indicate significant differences between different groups. Bar = 200 µm. The experiments were replicated six times with 1226 embryos.

To induce oxidative stress, H_2_O_2_ was used to treat porcine one-cell-stage parthenotes. When the parthenotes were exposed to 50 μM H_2_O_2_, the rates of blastocyst formation were significantly reduced (Fig. 2C, p < 0.01). Addition of 5 μg/mL CP provided notable protection as indicated by an increase in the percentage of embryos that reached the blastocyst stage compared to those treated with H_2_O_2_ alone (Fig. 2C, p < 0.01). The cleavage rate was not affected by H_2_O_2_ treatment alone or together with CP addition (Fig. 2B, p > 0.05).

Blastocysts were graded on a scale from 1 to 6 depending on their degree of expansion as shown in Fig. 2D. The rate of good-quality blastocysts (score 4–6) was significantly lower in the H_2_O_2_ treatment group than in the control, H_2_O_2_ + CP, and CP groups (Fig. 2E, p < 0.05).

### CP prevented H_2_O_2_-induced production of ROS

**Figure 3:**
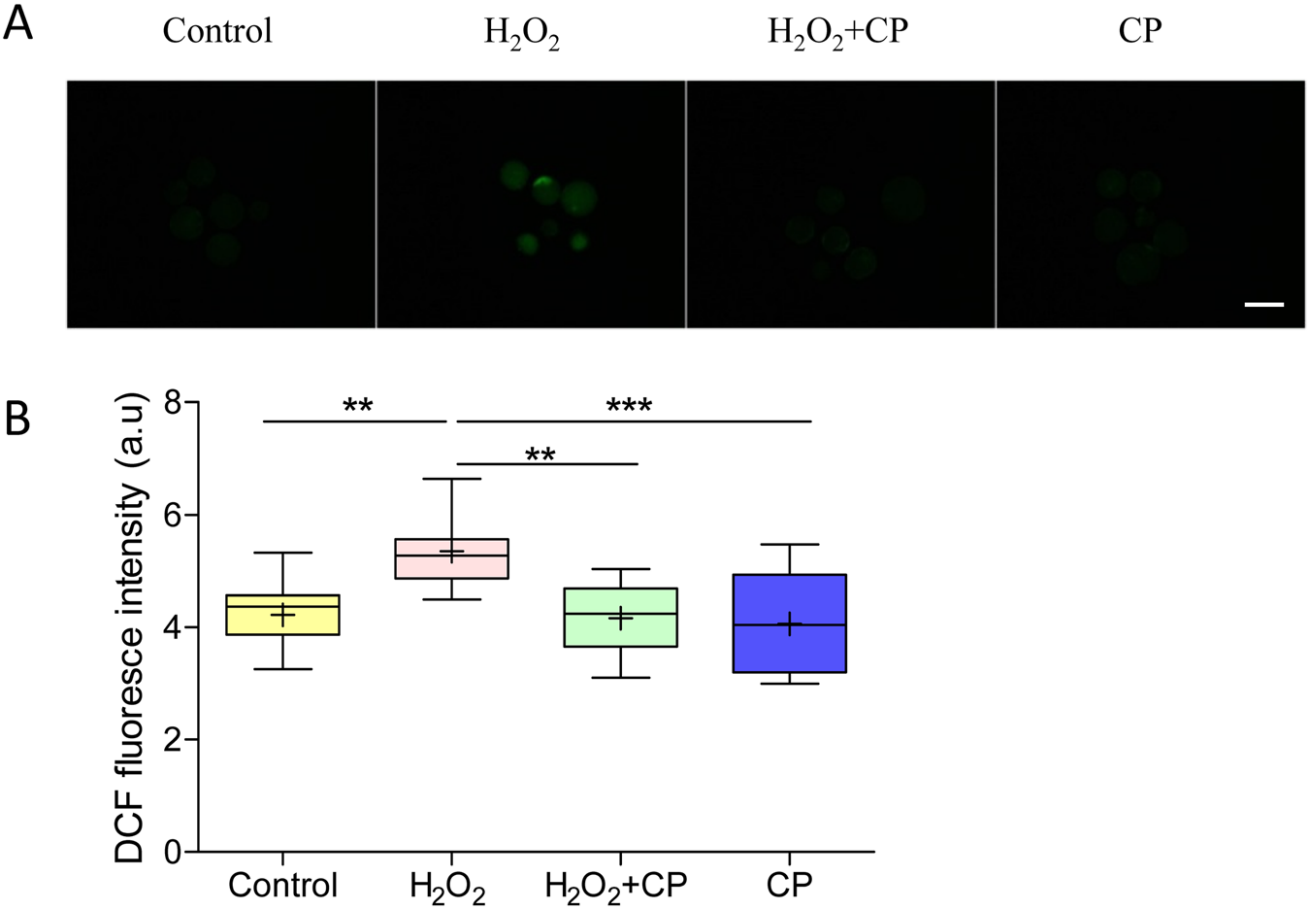
CP decreased H_2_O_2_-induced intracellular ROS level in early porcine blastocysts. (**A**) Fluorescence signals of ROS in blastocysts. (**B**) Intracellular ROS levels in blastocysts. Data are expressed as the mean ± SEM. In addition, **p < 0.01 and ***p < 0.001 indicate significant differences between different groups. Bar = 200 µm. The experiments were replicated three times with 187 blastocysts.

To determine antioxidation of CP in early porcine embryos, ROS level in blastocysts was measured using the DCFH fluorescent reaction. As shown in Fig. 3A, the DCFH fluorescence intensity was much higher in the H_2_O_2_-treated group compared with the control, H_2_O_2_ + CP, and CP groups. Quantification analysis showed that present of CP significantly decreased the level of intracellular ROS in blastocysts pre-treated with H_2_O_2_ (p < 0.01, Fig. 3B), suggesting that CP prevents H_2_O_2_-induced production of ROS.

### CP prevented mitochondrial dysfunction induced by H_2_O_2_ in porcine embryos

**Figure 4:**
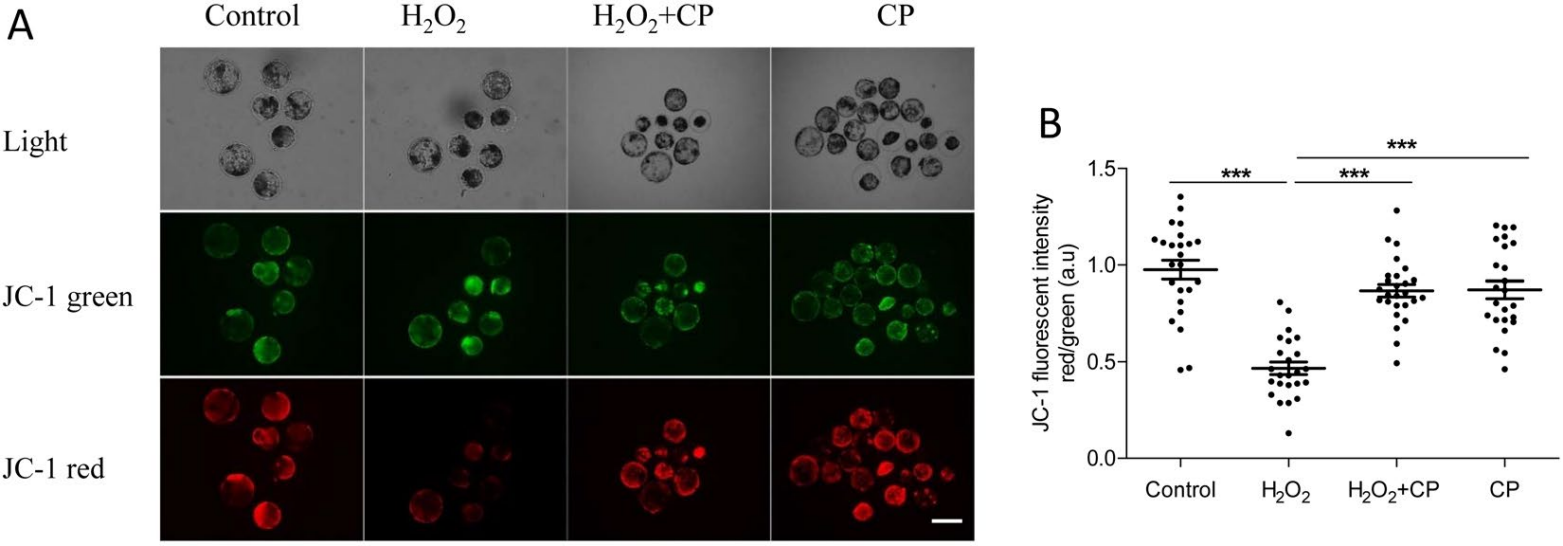
CP prevented H_2_O_2_-induced mitochondrial dysfunction in porcine blastocysts. (**A**) Representative fluorescence images of JC-1 staining in blastocysts. (**B**) Quantification of the ratio of fluorescence intensity (red/green) of JC-1 in blastocysts. Data are expressed as the mean ± SEM. In addition, ***p < 0.001 indicate significant differences between different groups. Bar = 200 µm. The experiments were replicated three times with 232 blastocysts.

Because mitochondria dysfunction is one of major reason that induces increase of ROS level and compromises embryo development^16,17^, we further explored mitochondrial membrane potential (MMP) in blastocysts using the JC-1 fluorescent reaction. As shown in Fig. 4A, H_2_O_2_ treatment enhanced green fluorescence and attenuated red fluorescence compared with that in the control, H_2_O_2_ + CP, and CP groups. Quantification analysis showed that the ratio of fluorescence intensity (red/green) decreased nearly 50% after H_2_O_2_ treatment, whereas it was recovered to levels similar to that in the control and CP alone groups in present of CP (p < 0.001, Fig. 4B).

### CP prevented H_2_O_2_-induced apoptosis

**Figure 5:**
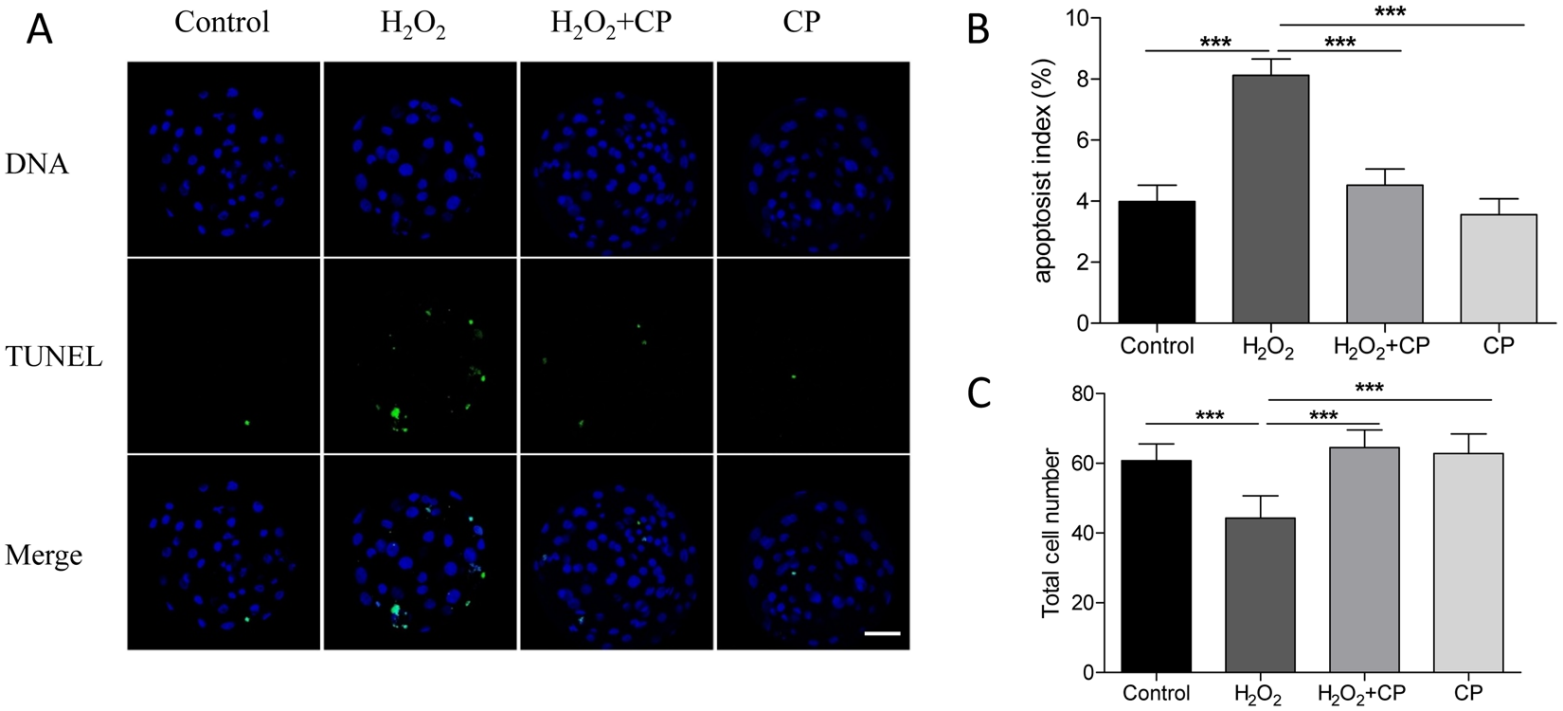
CP prevents H_2_O_2_-induced apoptosis in porcine blastocysts. (**A**) Representative fluorescence images of TUNEL staining in blastocysts. Apoptosis index (**B**) and total cell number (**C**) in blastocysts. Data are expressed as the mean ± SEM. In addition, *p < 0.05 and ***p < 0.001 indicate significant differences between different groups. Bar = 40 µm. The experiments were replicated four times with 334 blastocysts.

Apoptosis rate in blastocysts developed from H_2_O_2_-treated parthenotes in the presence or absence of CP was determined by TUNEL assay. As shown in Fig. 5, apoptosis rate of blastocyst pre-treated with H_2_O_2_ was significantly increased and the total cell number was decreased compared with control and CP alone groups (p < 0.001). However, blastocysts in the H_2_O_2_ + CP group had more cells than those treated only with H_2_O_2_ (p < 0.001). Furthermore, the number of TUNEL-positive cells decreased significantly in blastocysts derived from H_2_O_2_ + CP group as well (p < 0.001), suggesting a protective effect of CP against apoptosis in blastocysts.

### CP prevented H_2_O_2_-induced release of cytochrome c from mitochondria

**Figure 6:**
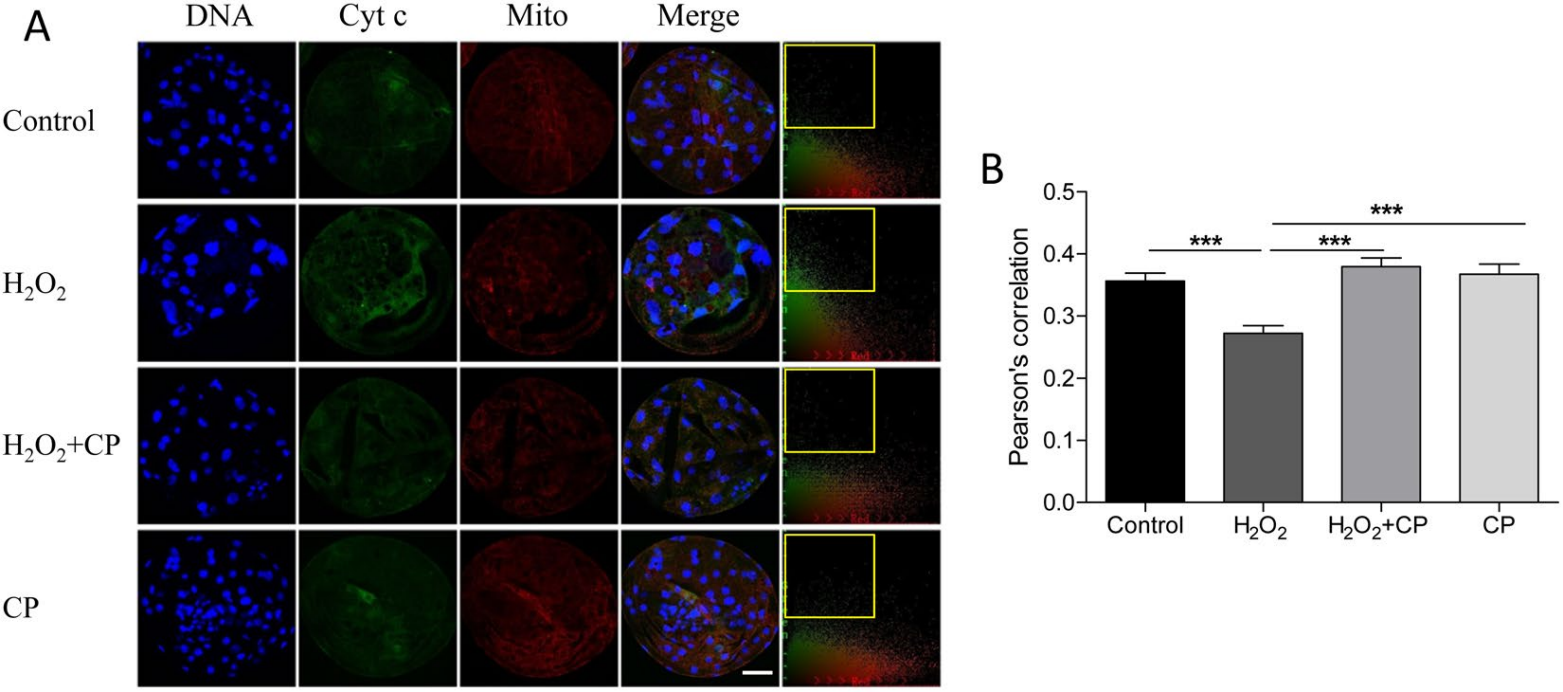
Colocalization of cytochrome c and mitochondria in blastocysts in different groups. (**A**) Blastocysts were labeled with a cytochrome c specific antibody (green) and Mito Tracker Red. (**B**) Colocalization was analyzed using Image-Pro Plus; frame indicates released cytochrome c. Pearson’s correlation was analyzed using Image-Pro Plus. Data are expressed as the mean ± SEM. In addition, ***p < 0.001 indicates significant differences between different groups. Bar = 40 µm. The experiments were replicated three times with 246 blastocysts.

To determine how CP decreased H_2_O_2_-induced apoptosis, colocalization of the mitochondria and cytochrome c was analyzed, as shown in Fig. 6. In H_2_O_2_-treated blastocysts, more cytochrome c was dispersed throughout the cytoplasm than in the control, H_2_O_2_ + CP, and CP groups. Pearson’s correlation showed that CP can prevent H_2_O_2_-induced cytochrome c release (p < 0.001).

### CP prevented H_2_O_2_-induced DNA damage

**Figure 7:**
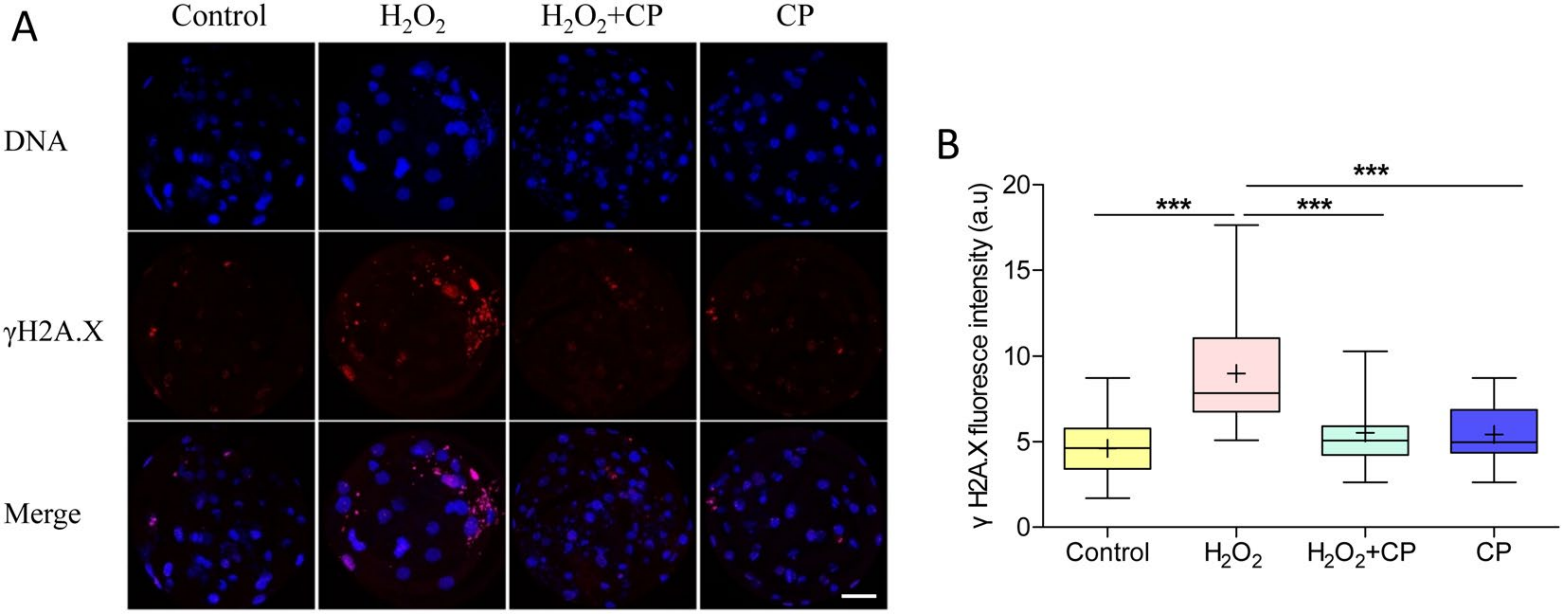
CP attenuated H_2_O_2_-induced DNA damage in porcine blastocysts. (**A**) Representative fluorescence images showing the presence of γH2A.X protein in the nuclei of porcine blastocysts. (**B**) Fluorescence intensity of γH2AX in blastocysts. Data are expressed as the mean ± SEM. In addition, ***p < 0.001 indicates significant differences between different groups. Bar = 40 µm. The experiments were replicated three times with 168 blastocysts.

The effect of CP on DNA damage (as indicated by γH2A.X) in porcine parthenogenetic embryos was shown in Fig. 7. Compared with control and CP groups, DNA damage in blastocysts pre-treated with H_2_O_2_ was much higher, whereas damage was markedly decreased in the presence of CP (p < 0.001).

### CP prevented H_2_O_2_-induced autophagy

**Figure 8:**
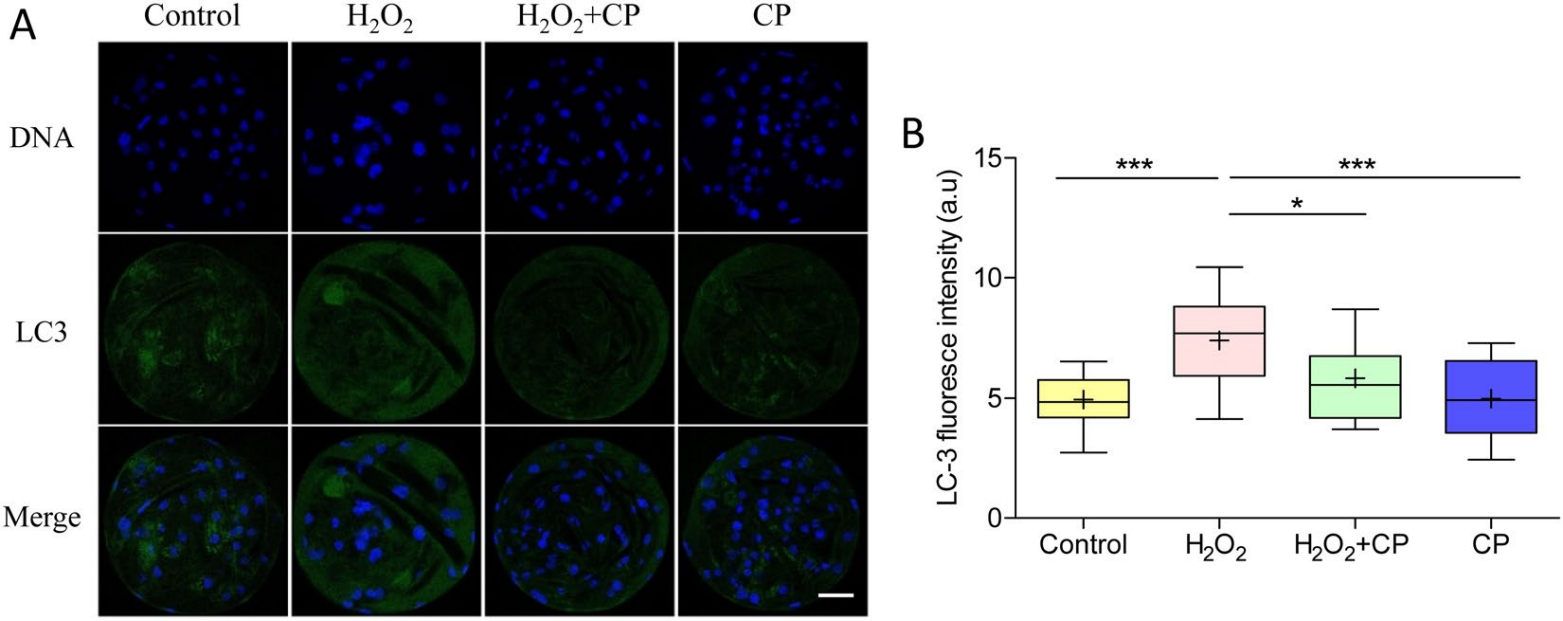
CP attenuated H_2_O_2_-induced autophagy in porcine parthenogenetic blastocysts. (**A**) Representative fluorescence images showing the presence of LC3 protein in porcine blastocysts. (**B**) Fluorescence intensity of LC3 in blastocysts. Data are expressed as the mean ± SEM. In addition, ***p < 0.001 indicates significant differences between different groups. Bar = 40 µm. The experiments were replicated three times with 205 blastocysts.

Oxidative stress can induce cell autophagy. Therefore, the expression level of LC3 was examined after treatment with or without H_2_O_2_ or CP. Immunofluorescence staining revealed a significant increase of LC3 in fluorescence intensity of H_2_O_2_-treated embryos (p < 0.001), which was markedly decreased in the presence of CP (Fig. 8, p < 0.05).

## Discussion

During *in vitro* production of preimplantation embryos, oxidative stress impairs embryo development and quality^18^. The present study showed that CP promoted the porcine embryo development from one-cell stage parthenotes to the blastocyst stage pre-treated with or without H_2_O_2_ and decrease apoptosis in blastocysts in present of H_2_O_2_, suggesting CP had a protective effect against oxidative stress on porcine preimplantation embryos.

Previous studies showed that CP has antioxidative capacity in several other cell types, including 2D and 3D astrocytes^19^, Madin-Darby canine kidney cells^8^, and chondrocytes cells^12^; however, the present study firstly evaluates the effect of CP on the development of early porcine embryos under oxidative stress. Our data showed addition of 5 μg/mL CP consumedly improved the development and quality of early porcine embryos, as indicated by increased blastocyst formation and hatching, reduced apoptosis, and less DNA damage and autophagy. These effects of CP on the development of preimplantation embryos may be related to the antioxidant and anti-apoptosis properties of CP in porcine embryos.

In biological systems, ROS are natural byproduct of the normal metabolism of oxygen and have important roles in cell of organism^20^. However, too much ROS caused by environmental stress impair cell structures, which finally induces apoptotic cell death in various cell types^21^. Previous study showed that CP notably bated apoptosis in Madin-Darby canine kidney cells by reducing intracellular ROS^8^. Moreover, CP also effectively prevented H_2_O_2_-induced ROS accumulation and inhibited apoptotic signals in 2D and 3D astrocytes^19^. Consistently, our data showed that supplementation of CP in culture media not only prevented production of ROS and decreased the number of apoptosis cells, but also significantly increased the total cell numbers in blastocysts.

In addition to preventing the direct production of ROS, maintaining stable mitochondrial function also appears to be a powerful antioxidant property of CP. During early mouse embryo development, mitochondrial dysfunction caused by treatment with H_2_O_2_ induce both cell cycle arrest and apoptosis^22^. MMP is an important parameter of mitochondrial function. Dissipation of MMP has been shown to promote cytochrome c release into the cytosol under oxidative stress. As a result, a series of caspase related pathway are activated^23^. In present study, we found that treatment with H_2_O_2_ led to depolarizing the MMP; this effect was prevented by treatment with CP. These results are consistent with those of previous studies^8,24^. Furthermore, to detect whether apoptosis occur in porcine blastocysts was caused by releasing of cytochrome c from mitochondria, we also tested the colocalization of mitochondria and cytochrome c. Our results clearly revealed that mitochondria and cytochrome c did not colocalize in the H_2_O_2_ treatment alone group, indicating that cytochrome c was released into the cytosol. However, CP prevented H_2_O_2_-induced cytochrome c release.

DNA damage is another harmful effect of oxidative stress because DNA damage can induce apoptosis in several cells^3,25^. In the present study, DNA damage occurred in porcine blastocysts derived from H_2_O_2_ treated parthenotes, while CP prevented this H_2_O_2_-induced DNA damage. This may explain how CP attenuated oxidative stress-induced apoptosis.

Autophagy, a natural, regulated mechanism of the cell that degrades unnecessary proteins and dysfunctional organelles, represents a general cellular and tissue response to oxidative stress^26,27^. Under oxidative stress, chaperone-mediated autophagy was activated and oxidized proteins with higher susceptibility was removed to protect the cells from ROS-induced damage^28^. However, when oxidative stress reaches a level beyond control of protective respond of autophagy, cell death occurs through necrosis, apoptosis, or autophagic cell death^29,30^. In the present study, the autophagy marker LC3 was used to estimate the level of autophagy in porcine blastocysts. H_2_O_2_-induced oxidative stress increased autophagy and apoptosis in porcine blastocysts, whereas autophagy and apoptosis in porcine blastocysts in the control, H_2_O_2_ + CP, and CP groups with low oxidative stress were lower. Furthermore, apoptosis and autophagic cell death under high oxidative stress levels may contribute to a low total cell number in present of H_2_O_2_. The protective effect of CP increased the total cell number by preventing oxidative stress.

**Figure 9:**
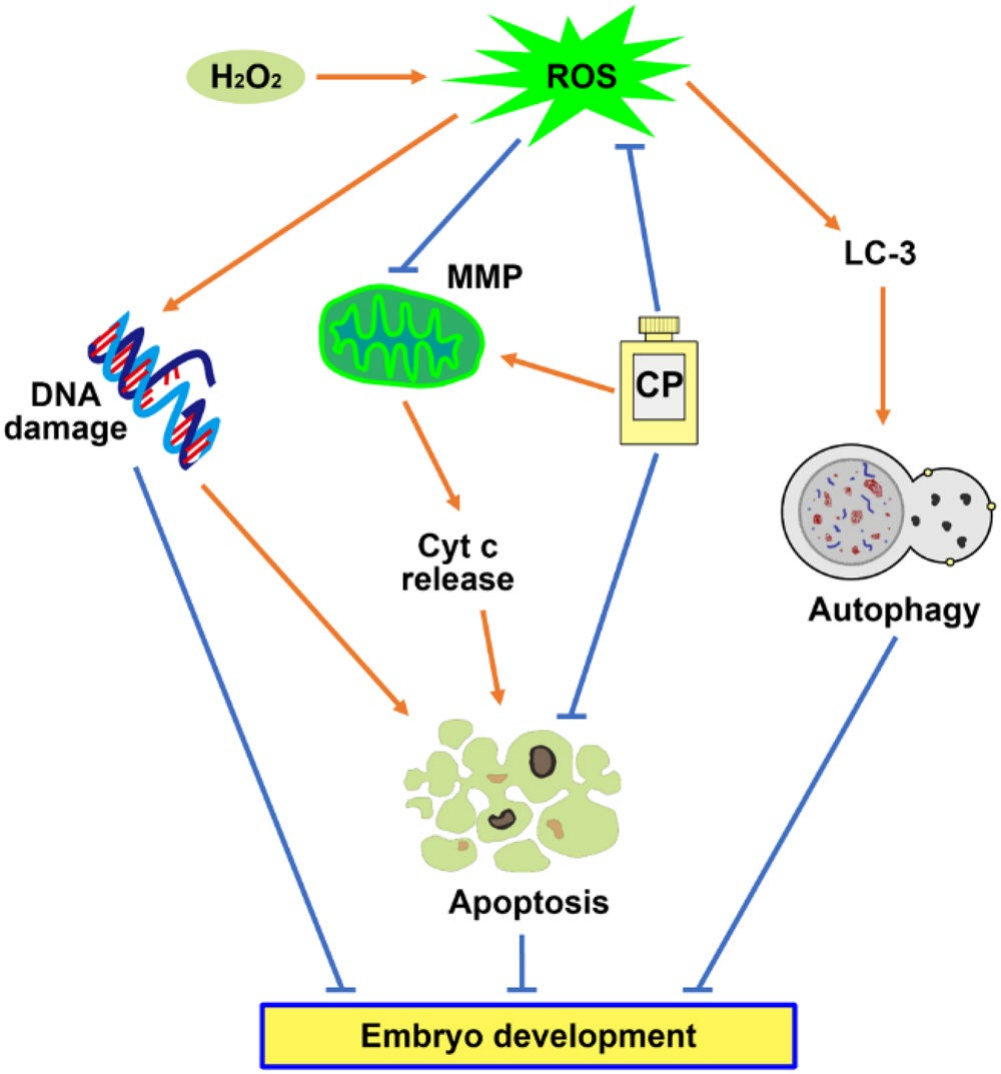
Schematic representation of the protective effect of CP during porcine embryonic development. H_2_O_2_ exposure causes the generation of oxidative stress, resulting in DNA damage, autophagy, and mitochondrial dysfunction. Apoptosis was induced by the release of cytochrome c from the mitochondria because of the compromise of MMPs. Addition of CP promotes embryo development by attenuating H_2_O_2_-induced oxidative stress, apoptosis, and compromised MMP because of the antioxidative, anti-apoptosis, and protection mitochondrial dysfunction properties.

In conclusion, although embryonic cleavage was not affected by addition of CP, 5 μg/mL CP significantly increased blastocyst formation and the hatching rate during early porcine embryo development. As shown in Fig. 9, CP prevented H_2_O_2_-induced production of ROS and depolarization of MMP and release of cytochrome c from the mitochondria. Furthermore, apoptosis, DNA damage level, and autophagy in the blastocysts were attenuated by supplementation with CP after H_2_O_2_ treatment. Taken together, these results suggest that CP has beneficial effects on the development of porcine parthenotes by attenuating mitochondrial dysfunction and oxidative stress.

## Materials and Methods

All chemicals were purchased from Sigma-Aldrich Co., Inc. (St. Louis, MO, USA) unless otherwise indicated. All manipulations were performed on a heated stage adjusted to 38.5 °C unless otherwise indicated.

### Collection of porcine oocytes and *in vitro* maturation

Ovaries from pre-pubertal gilts were collected from a local slaughterhouse (Farm story dodarm B&F, Umsung, Chungbuk, Korea) and transported to the laboratory at 37 °C in saline supplemented with 75 mg/mL penicillin G and 50 mg/mL streptomycin sulfate. Follicles 3–6 mm in diameter were aspirated. Cumulus–oocyte complexes were selected according to their morphologic characteristics, i.e., those showing at least three layers of compact cumulus cells and evenly granulated ooplasm. Briefly, the COCs were washed three times with TL-HEPES supplemented with 0.1% polyvinyl alcohol (PVA, w/v) and 0.05 g/L gentamycin. Next, the COCs were washed three times in maturation medium (TCM-199 supplemented with 0.1 g/L sodium pyruvate, 0.6 mM L-cysteine, 10 ng/mL epidermal growth factor, 10% porcine follicular fluid, 10 IU/mL luteinizing hormone, and 10 IU/mL follicle-stimulating hormone) and then transferred to maturation medium. Maturation was performed by culturing approximately 50 COCs in 500 μL of maturation medium in 4-well dishes. The medium was covered with mineral oil and the plates were incubated at 38.5 °C in a humidified atmosphere of 5% CO_2_ for 44 h.

### Parthenogenetic activation and *in vitro* culture

After removing cumulus cells by repeated pipetting in 1 mg/mL hyaluronidase, denuded oocytes were parthenogenetically activated by 2 direct-current pulses of 120 V for 60 µs in 297 mM mannitol (pH 7.2) containing 0.1 mM CaCl_2_, 0.05 mM MgSO_4_, 0.01% PVA (w/v), and 0.5 mM HEPES. These oocytes were cultured in bicarbonate-buffered porcine zygote medium 5 (PZM-5) containing 4 mg/mL BSA and 7.5 µg/mL cytochalasin B (CB) for 3 h to suppress extrusion of the pseudo-second polar body. Next, the oocytes were thoroughly washed and cultured in bicarbonate-buffered PZM-5 supplemented with 4 mg/mL BSA in 4-well plates for 7 days at 38.5 °C and 5% CO_2_. To determine the dose-effect of CP on early porcine embryos cultured *in vitro*, the activated embryos were cultured with CP at various concentrations (2, 5, 8, 10 µg/mL). In addition, to determine the protective effect of CP against oxidative stress, the activated embryos were pre-incubated with CP for 3 h. After pre-incubation, H_2_O_2_ (50 µM) was added for 30 min. Then, the embryos were washed three times and transferred into PZM-5 medium with or without CP. Cleavage and blastocyst formation rates were examined on days 2 and 7 after activation. After 7 days of culture, the quality of the blastocysts was evaluated according to Gardner’s criteria^31^. Blastocysts were graded on a scale from 1 to 6 depending on their degree of expansion as follows: Grade 1, Blastocyst cavity occupies less than half of the blastocoel volume of the embryo; Grade 2, Blastocyst cavity occupies more than half of the blastocoel volume of the embryo; Grade 3, Blastocyst cavity completely fills the embryo; Grade 4, Blastocyst cavity occupies more than the blastocoel volume of the embryo, with a thinning zona pellucida; Grade 5, Hatching out of the zona pellucida; Grade 6, Hatched out of the zona pellucida. To determine the total cell number, day 7 blastocysts were randomly collected and stained with 10 mg/mL Hoechst 33342 in PBS for 5 min.

### Measurement of ROS contents

ROS in blastocysts were detected using 2′,7′-dichlorodihydrofluorescein diacetate (H2DCF-DA, Cat #D399, Molecular Probes Invitrogen, Eugene, OR) as previously described^32^. Briefly, blastocysts were incubated for 15 min in PBS/PVA containing 10 µM H2DCF-DA at room temperature. After incubation, blastocysts were washed three times with PBS/PVA. Fluorescent signals were captured as a TIFF file using a digital camera (DP72; Olympus, Tokyo, Japan) connected to a fluorescence microscope (IX70, Olympus). Quantification of ROS levels was performed by analyzing the fluorescence intensity in blastocysts using Image J version 1.44 g software (National Institutes of Health, Bethesda, MD, USA).

### Terminal deoxynucleotidyl transferase-mediated 2′-deoxyuridine 5′-triphosphate (dUTP) nick-end labeling (TUNEL) assay

The intracellular apoptosis level of blastocysts was measured in a TUNEL assay using an *in situ* cell death detection kit (Cat #11684795910, Roche) as described previously^33^. After washing three times with PBS/PVA, blastocysts were fixed in 3.7% paraformaldehyde for 30 min at room temperature and subsequently permeabilized by incubation in 0.5% Triton X-100 for 30 min at room temperature. The embryos were incubated with fluorescein-conjugated dUTP and terminal deoxynucleotidyl transferase enzyme for 1 h, and then washed three times with PBS/PVA. Embryos were treated with 10 µg/mL Hoechst 33342 for 5 min, washed three times with PBS/PVA, and mounted onto glass slides. Images were captured using a confocal microscope (Zeiss LSM 710 META, Jena, Germany). The apoptosis index was calculated as the percentage of TUNEL-positive nuclei.

### Immunofluorescence and confocal microscopy

After washing three times with PBS/PVA, embryos were fixed in 3.7% paraformaldehyde for 30 min at room temperature, permeabilized with PBS/PVA containing 0.5% Triton X-100 at room temperature for 30 min, and incubated in PBS/PVA containing 1.0% bovine serum albumin at room temperature for 1 h. These embryos were incubated overnight at 4 °C with anti-LC3 (1:100; Cat #ab58610, Abcam, Cambridge, UK), anti-cytochrome c (1:100; Cat #ab110325, Abcam, Cambridge, UK), and anti-γH2A.X (1:100; Cat #2577, Ser139, Cell Signaling Technology, Danvers, MA, USA) diluted 1:100 in blocking solution. After washing three times with PBS/PVA, the embryos were incubated at room temperature for 1 h with goat anti-rabbit IgG, rabbit anti-goat IgG, or anti-mouse IgG. The oocytes and embryos were stained with 10 µg/mL Hoechst 33342 for 5 min, washed three times with PBS/PVA, mounted onto slides, and examined using a confocal microscope (Zeiss LSM 710 META). Images were processed using Zen software (version 8.0, Zeiss).

### Assay of mitochondrial membrane potential (∆Ψm)

Day-7 blastocysts were incubated in PZM-5 containing 2.5 µM 5,5′,6,6′-tetrachloro-1,1′,3,3′-tetraethyl-imidacarbocyanine iodide (JC-1) (Cat #M34152, Invitrogen, Carlsbad, CA, USA) at 38.5 °C in 5% CO_2_ for 30 min. Membrane potential was calculated as the ratio of red florescence, which corresponded to activated mitochondria (J-aggregates), to green fluorescence, which corresponded to less-activated mitochondria (J-monomers)^34^. Fluorescence was visualized using an epifluorescence microscope (Nikon Corp., Tokyo, Japan).

### Colocalization assay of mitochondria and cytochrome c

To investigate the colocalization of mitochondria and cytochrome c, blastocysts were incubated with 500 nM MitoTracker Red CMXRos (Cat #M7512, Invitrogen, Eugene, OR, USA) at 38.5 °C for 30 min. After three washes with PZM-5, staining of cytochrome c was carried out as described in **Immunofluorescence and confocal microscopy**.

### Real time reverse transcriptase-polymerase chain reaction (RT-PCR)

Day-7 blastocysts were collected and mRNA was extracted from a pool of 10 blastocysts per group using the DynaBeads mRNA Direct Kit (Cat #61012, Dynal Asa, Oslo, Norway) according to the manufacturer’s instructions. cDNA was obtained by reverse transcription of mRNA using the Oligo (dT)12–18 primer and SuperScript III Reverse Transcriptase (Invitrogen). Amplification was conducted as follows: 95 °C for 3 min followed by 40 cycles of 95 °C for 15 s, 60 °C for 30 s, and 72 °C for 20 s, and final extension at 72 °C for 5 min. Target genes were *COX2*, *FN1*, and *ITGA5*. *GAPDH* was used as a reference gene. The primers used to amplify each gene are shown in Table 1 ^35^. mRNA quantification data were analyzed using the 2^−ΔΔCT^ method^36^.

**Table 1:** Primer sequences used in real time RT-PCR.

Gene	Primer sequence (5′-3′)	Product Size (bp)
*COX2*	F:GGCTGCGGGAACATAATAGA	183
	R:GCAGCTCTGGGTCAAACTTC	
*FN1*	F:AGGGCGATGAACCACAGT	221
	R:GCTCCAGCGAACGACAAT	
*ITGA5*	F:TGATGACAGTTATTTGGGCTAC	100
	R: CAAAGTCCTCGCTGCTCT	
*GAPDH*	F:TTCCACGGCACAGTCAAG	117
	R:ATACTCAGCACCAGCATCG	

F: forward; R: reverse.

### Statistical analysis

All data were subjected to one-way analysis of variance. Differences among treatments were examined using the Duncan multiple range test. All percentage data were subjected to arcsine transformation prior to statistical analysis and presented as the mean ± SEM. Significance was set at p < 0.05. All calculations were performed using SPSS software v.19 (SPSS, Inc., Chicago, IL, USA). All box plots show the median (line), mean (+), and 25th and 75th percentiles (boxes) and the whiskers show the minimum to maximum values.

### Data Availability

All data generated or analysed during this study are included in this published article.
